# Towards a Scalable Software Defined Network-on-Chip for Next Generation Cloud

**DOI:** 10.3390/s18072330

**Published:** 2018-07-18

**Authors:** Alberto Scionti, Somnath Mazumdar, Antoni Portero

**Affiliations:** 1 Istituto Superiore Mario Boella (ISMB), 10138 Torino, Italy; scionti@ismb.it; 2 Simula Research Laboratory, 1325 Lysaker, Norway; mazumdar@simula.no; 3 IT4Innovations, VSB-University of Ostrava, 70833 Ostrava–Poruba, Czech Republic

**Keywords:** many-core, software-defined NoC, data-driven

## Abstract

The rapid evolution of Cloud-based services and the growing interest in deep learning (DL)-based applications is putting increasing pressure on hyperscalers and general purpose hardware designers to provide more efficient and scalable systems. Cloud-based infrastructures must consist of more energy efficient components. The evolution must take place from the core of the infrastructure (i.e., data centers (DCs)) to the edges (Edge computing) to adequately support new/future applications. Adaptability/elasticity is one of the features required to increase the performance-to-power ratios. Hardware-based mechanisms have been proposed to support system reconfiguration mostly at the processing elements level, while fewer studies have been carried out regarding scalable, modular interconnected sub-systems. In this paper, we propose a scalable Software Defined Network-on-Chip (SDNoC)-based architecture. Our solution can easily be adapted to support devices ranging from low-power computing nodes placed at the edge of the Cloud to high-performance many-core processors in the Cloud DCs, by leveraging on a modular design approach. The proposed design merges the benefits of hierarchical network-on-chip (NoC) topologies (via fusing the ring and the 2D-mesh topology), with those brought by dynamic reconfiguration (i.e., adaptation). Our proposed interconnect allows for creating different types of virtualised topologies aiming at serving different communication requirements and thus providing better resource partitioning (virtual tiles) for concurrent tasks. To further allow the software layer controlling and monitoring of the NoC subsystem, a few customised instructions supporting a data-driven program execution model (PXM) are added to the processing element’s instruction set architecture (ISA). In general, the data-driven programming and execution models are suitable for supporting the DL applications. We also introduce a mechanism to map a high-level programming language embedding concurrent execution models into the basic functionalities offered by our SDNoC for easing the programming of the proposed system. In the reported experiments, we compared our lightweight reconfigurable architecture to a conventional flattened 2D-mesh interconnection subsystem. Results show that our design provides an increment of the data traffic throughput of 9.5% and a reduction of 2.2× of the average packet latency, compared to the flattened 2D-mesh topology connecting the same number of processing elements (PEs) (up to 1024 cores). Similarly, power and resource (on FPGA devices) consumption is also low, confirming good scalability of the proposed architecture.

## 1. Introduction

Cloud-based execution environments are in place to process the complex machine learning (ML) algorithms. Microsoft’s project Catapult and Google’s TPU (custom ASICs designed for Deep learning algorithms) are a few examples of increasing Cloud support for efficient processing of the ML-based application. Deep learning [1] (DL—essentially a subset of ML) is very useful in solving complex problems where human or artificial thought is required. Currently, there is a flurry of research for designing more efficient DL-based algorithms and custom hardware accelerators to execute them better (such as the Xilinx reconfigurable acceleration stack). Most of these accelerators are spatial (i.e., an array of interconnected PEs), with input data elaborated following a data-driven approach. In general, a considerable research effort has been spent to improve the micro-architecture of PEs, while ignoring the interconnection subsystem. Most of the ML-based accelerators are specialised buses, mesh-based, or crossbars. On the other hand, the availability of billions of transistors in modern silicon devices provides room for implementing more efficient interconnections and execution mechanisms, supporting massive parallelism. However, to fully unleash the potential benefits of such kind of architectures, an easy and effective way of controlling reconfiguration is paramount.

Modern silicon technology makes it possible to wrap thousands of PEs on a chip [2]. The massive number of PEs provides the execution capability for executing an enormous number of concurrent threads. Current designs implement a large number of single-issue, in-order cores to limit the power dissipation [3]. Such methodology consumes less energy than their multi-issue, out-of-order counterparts [4,5]. Another way to improve the performance and the energy efficiency is the exploitation of hardware–software monitors, which keep track of resource usage over time. They are very helpful to capture the behaviour of parallel applications. Although these techniques contribute to better parallelism exploitation (especially in the context of massive many-core CMPs), the use of classical Von Neumann program execution models (PXMs) quickly becomes the bottleneck. In multi-threaded applications with several thousands of concurrent threads, Von Neumann PXMs create a large synchronisation overhead as well as uncertainty around accuracy and race condition free execution [6]. Conversely, PXMs that are based on an explicit data-driven execution (e.g., the Codelet model [7]) allow for self-scheduling and isolation of execution properties. These properties help in limiting the synchronisation overhead and allow the application to grow and shrink over time. Furthermore, data-driven PXMs offer a valuable means for efficiently running DL algorithms, which are data-driven in nature. Despite the benefits, adopting such PXMs brings a major challenge in the interconnect subsystem.

Software-defined networks (SDNs) is a relatively new concept that is used in several fields like in wireless sensor architectures [8,9], fifth-generation cellular systems (5G) [10,11], future smart systems [12] and cognitive routing [13]. SDN is becoming popular because of its flexibility to separate the hardware packet forwarding mechanism from the control logic [14,15]. SDN allows us to integrate network control mechanisms with application software, thus facilitating execution of the application and providing better network support. In this work, we focus on applying the SDN paradigm to the management of NoC, thus aiming at providing a more flexible and efficient solution for supporting new Cloud supported ML applications.

In this paper, we focus on the interconnect subsystem, proposing a scalable fabric that merges the advantages of ring-based interconnects (for the local data packet communication) with those carried out by the reconfiguration and a global 2D-mesh based interconnection. The partial idea of the paper is fundamentally influenced by [16]. Hierarchical organisation of the network allows for better exploitation of data and computation locality. We introduced a way to dynamically change the topology of the network itself, although the physical organisation of the fabric uses a fixed topology. A small group of instructions is added to the PEs’ ISA to allow the software directly controlling the network during the execution. We refer to our architecture as a SDNoC [17]. With applications in which operations are hierarchically grouped and dynamically scheduled [7], our adaptation allows better exploitation of the network resources and more significant power savings. Using hierarchical topology based on local rings attached to mesh routers, our design allows the software to monitor and control links with fine granularity. As a result, network resources can be switched off when not used. Link usage is monitored, and internal hardware counters are read via the dedicated instructions. This information can be exploited by optimisation tools and compilers to better adapt to the communication patterns of an application. Our proposed approach aims at solving three primary challenges, such as flexibility, scalability and interoperability, leaving out security aspects [18]. Our customised architecture can route the packets as per the compiler level instructions. Productivity is also improved by introducing data-driven PXM support into a high-level programming language, thus allowing the application developer to readily exploit the benefit of interconnection reconfigurability and data-driven execution.

The remainder of the paper is organised as follows. Section 2 provides an overview of the proposed reconfigurable NoC, by describing its hierarchical organisation. The most relevant works are also reviewed and the paper contribution is clearly stated in its subsections. Next, the primary architecture of the proposed interconnection fabric is presented in Section 3. Micro-architecture both of the router and the ring switch is presented along with the control flow technique used to manage the traffic. Section 4 describes the architectural extensions of the NoC to provide the needed adaptability (reconfiguration). Specifically, the communication protocol implemented to support PE-to-PE data exchange is discussed. It is followed by Section 5, where the programming interface is analysed. Furthermore, to ease the development of applications, a high-level programming interface is also described, along with the low-level set of additional PE’s instructions, as well as the corresponding execution model. Section 6 provides the experimental evidence of the advantages (in terms of performance, area and power consumption) brought by the proposed design compared to the flattened 2D-mesh. Finally, Section 7 presents some future work directions and concludes the paper.

## 2. System Overview

Tile-based PE organization enables a modular design [19]. We propose a coarse-grain reconfigurable architecture (CGRA) for supporting emerging applications (e.g., DL) through an energy-efficient computing sub-system that encompasses a large number of computing tiles. At the edge of Cloud infrastructures, nodes require moderate computing capabilities, while limiting power consumption becomes a more pressing issue. A reduced number of tiles, equipped with an adequate number of I/O interfaces, support better acquisition and can ingest a large amount of data. Conversely, infrastructures’ cores are based on more powerful computing chips. In such scenarios, performance requirements dominate, albeit keeping power consumption under control is still paramount. The highest parallelism is helpful in supporting applications with high demands for bandwidth and processing capabilities. To this end, each tile contains several PEs and I/O units organized into small clusters. Each cluster is directly interconnected to a 2D-mesh router (R). PE’s architecture may vary, depending on the specific application requirements, ranging from small in-order cores to tiny and very optimized DSP-like elements. While the former provide the best trade-off between computing capability, energy efficiency and flexibility (i.e., better support for general purpose applications), the latter can be optimized for specific applications domains, whereas the type of operations to perform is restricted. From this viewpoint, the proposed interconnection is agnostic with regards to the PE architecture. It only requires the extension of the standard ISA with a small set of additional instructions to control the flow of data inside the fabric. In addition, a local software-managed scratchpad memory is available to each PE.

**Figure 1:**
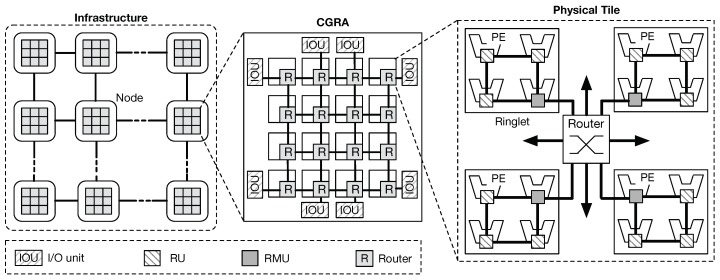
Overview of the proposed scalable network-on-chip fabric. Multi-node configurations exploit the availability of high-speed I/O units.

**Figure 2:**
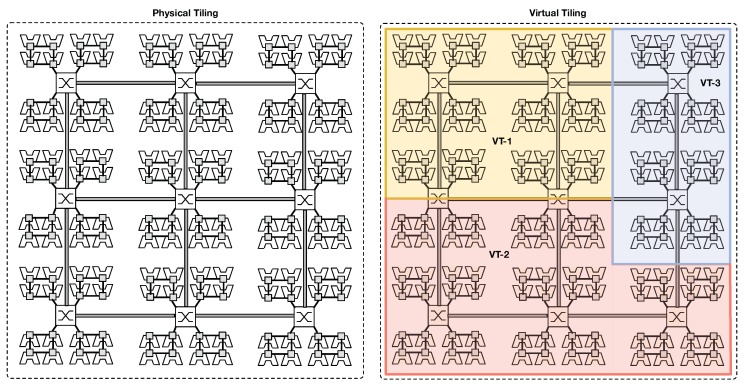
Mapping of virtual tiles (VT-*i*) on the physical chip. For instance, three applications/tasks are mapped on the chip (VT-1, VT-2 and VT-3), each using a reserved portion of the hardware resources.

Figure 1 shows an instantiation of the proposed CGRA architecture. Distributed nodes, each equipped with the proposed fabric design, are interconnected each other to form a single distributed cluster. Within each node, the CGRA is composed of several tiles (physical tile), providing connectivity and processing power. PEs are organized into groups interconnected through a bi-directional ring, referred to as *ringlets*. While rings provide high-speed low-latency connectivity for local communications, mesh routers provide global connectivity. Interconnection between ringlets and the routers is obtained by extending the network interface of one of the units in the ring (RUs) with extra-logic. Such units are referred to as *ring-master units* (RMU). On the other hand, routers form a physical 2D-mesh network topology, providing enough bandwidth for supporting less frequent chip-level communications. Such topology has become very popular in complex many-core designs [19], thanks to a better offering in terms of wiring area, power cost, and fault tolerance [20]. By adding extra logic in the router micro-architecture, our design enables the software application to generate virtual topologies and partition resources on a per task basis (*virtual tiles*), over the physical substrate. In fact, the combination of these two physical topologies allows our scalable design to result in a higher throughput capacity, lower communication latency, and better energy efficiency, contrasting with conventional flattened 2D-mesh designs (i.e., local rings consume less power respect to 2D-mesh routers, although they serve more frequent packet exchanges). Figure 2 depicts an example of virtual tile creation over the physical fabric. Furthermore, software dynamic reconfiguration of the underlying physical fabric allows for better accommodating different application communication patterns, still exploiting the fact that most of the communication in a parallel application affects a group of resources (i.e., PEs and routers) that are close to each other [21]. Since the larger portion of the interconnection is represented by rings, the area cost can also be reduced by our approach. Finally, massive parallelism exploitation is guaranteed by the small instruction set extension (ISE) embedded in each PE, which explicitly target a data-driven PXM. In this execution model, the application is divided into a set of fine-grain tasks, each exchanging data with others through an explicit producer–consumer scheme. In order to preserve locality and hide latency, tasks can be grouped to form small task-graph functions scheduled for execution on PEs that are close to each other.

To ease the programming of the proposed architecture, we introduce a mechanism to map a high-level programming language to the data-driven PXM supported by our SDNoC. Specifically, a programming language (e.g., Go, P4 [22]) that inherently implements parallel execution mechanisms mapped on low-level functionalities offered by our architecture, (i.e., instructions to control the underlying reconfigurable hardware). Since the proposed architecture is designed to be agnostic regarding the specific PE micro-architecture, they can be further specialised to provide hardware implementation for specific functions (e.g., network packet parsing, packet filtering).

### 2.1. Challenges and State-of-the-Art

The growing number of services and new applications running on the Cloud are ever more performance and energy hungry. To this end, Cloud service providers also offer services with accelerated instances. Although GPUs are the most common accelerator candidates, other types of accelerators are emerging, and their adoption at scale is growing. For instance, special purpose devices used to accelerate DL algorithms integrated into hyper-scale computing infrastructures (such as the case of Google TPUs). Public Cloud service providers (such as Amazon, Microsoft) offer the computational facilities that leverage on field programming gate arrays (FPGAs) to deliver high energy efficiency. FPGAs are primarily used to implement inference accelerators. There are still several technical challenges needed to be addressed to enable a massive diffusion and ease of use of such heterogeneous devices. The software integration is even a more significant challenge since there are several programming frameworks (e.g., TensorFlow, PyTorch) and libraries (e.g., Apache MLlib) available. Often, supporting a new hardware device requires a significant effort to rewrite compilers back-end to enable users to use such frameworks and libraries. On the other hand, there is a demand for a careful design of the interconnection subsystem of hardware accelerators to provide enough bandwidth and reduced latency.

Overcoming such challenging issues requires designing scalable, reconfigurable systems, which can be dynamically configured depending on the specific requirements of the software application. Furthermore, high-level programming languages must be extended to control the system and dynamically handle its reconfiguration process. Compiler back-end must be provided to correctly target the specific instruction sets, as well as to expose to the programming language an abstracted view of the underlying hardware. Furthermore, programming languages should provide programming structures and semantics to improve the parallelism. Some efforts are made in creating domain specific languages (DSLs) that can incorporate desired features for better execution support to applications of specific domains. For instance, P4 is proposed as a way to program custom accelerators for network processing and traffic filtering. It is designed to be agnostic with regards to the hardware substrate. However, a natural target for the language compiler are FPGA-based platforms.

Flattened 2D-mesh is a very popular topology for many-core CMPs, but it presents three significant criticalities to scalability. Firstly, with the increasing number of connected tiles, the NoC quickly consumes a significant portion of the overall power budget. Secondly, with the increase in the number of concurrent tasks, the network bandwidth is rapidly consumed. Finally, fixed network topology neither supports the principle of locality exploited by hierarchical data-driven PXMs well nor does it adapt to the changes in the communication patterns. Tackling these criticalities requires the network to be dynamically configured. Selectively switching off links that are not used allows the network to save power. Previous works focused only on specific aspects. Kim et al. [23] propose a scalable 2D-mesh, where routers are implemented through lightweight ring-based switches. Although the simpler micro-architecture allows routers to scale better and consume less power (energy), the topology of the network remains fixed, thus lightly used links cannot be excluded. Conversely, Panthre [24] integrates reconfiguration features within a conventional router micro-architecture. Despite power saving benefits, routers are based on the conventional micro-architecture. Such an approach is inherently power hungry since the majority of the power is dissipated through the crossbar switch and link drivers. Other works, e.g., [25], is focused on the analysis of fixed hybrid topologies. In [16], the authors presented their approach to software programming of the NoC topology. Here, the physical substrate is based on a 2D-mesh network, built on top of simple rings running in both the *x*- and *y*-dimensions of the mesh. Ring stations have been augmented to enable traffic steering from one dimension to the other, while ISA of PE has been extended to control network configuration and dispatching application threads. This work is more in line with our proposed architecture, and, despite its good scalability, the evaluation on the area consumption is lacking. In [26], the authors proposed a hybrid software-hardware NoC architecture. In contrast to conventional NoCs, the control plane is implemented as a software module running on a dedicated core. In this work, the core (i.e., the actual network manager) can take dynamic decisions on the network configurations. Such a solution can easily adapt to irregular traffic patterns depending on the current state and application demands. However, it still relies on a centralised component that could quickly become the bottleneck and limit the overall scalability.

In [22], the authors propose how to control the packet processing while making the switches agnostic to the hardware implementation. The programmer can decide the functionality to process the packets. Next, a compiler transforms an imperative program into a table dependency graph that can be mapped to multiple specific target switches. This approach first sets the functionality of the switches and then tells how the packets need to be processed. In another work [27], protocol-oblivious forwarding (POF) based on a generic flow instruction set for programmable SDNs is proposed. The primary idea of this work is to remove the dependency on protocol-specific configurations on the forwarding elements and also enhance the data-path with new stateful instructions. The author claims that the approach can reduce the network cost by using commodity forwarding elements. The open packet processor (OPP) [28] is proposed to improve the interaction between platform-independent HW configurability and packet-level programming flexibility. It aims at helping programmers to deploy stateful forwarding tasks. The authors claim that the proposed approach shows the viability of extended finite state machines (FSMs) as low-level data plane programming abstraction together with stateful operation and flow-level feature tracking.

### 2.2. Paper Contribution

The main contributions of the paper are as follows. Starting from the design presented in [29], we extended the original architecture up to 1024 PEs. Performance and area/power costs are evaluated on a more extensive and complex configuration. The proposed design merges the benefits of a lightweight and scalable hybrid interconnection, based on the 2D-mesh routers and local rings, with those of the dynamic reconfiguration. In the proposed hierarchical topology, routers are in charge of moving traffic at a global (chip) level, while exploiting data locality is ensured by groups of PEs connected to each other through fast and low power bi-directional rings. Router micro-architecture has been extended to support dynamic reconfiguration (virtual tiles). To this end, groups of specific bits control the status of each link. During the configuration phase, the links can be power gated (PG). PG mode disables the links, thereby allowing the network to save power. Furthermore, PG mode allows for better use of hardware resources. Figure 2 shows an example of such resource splitting. The left side of the figure shows the physical chip infrastructure, while the right side depicts an example of virtual tiling (i.e., three applications/tasks concurrently run on the chip using reserved resources). Eventually, virtual tiles can span on multiple chips, thanks to the use of dedicated I/O units.

Performance can be assured by using high-speed clocks in the rings, while monitoring is provided by a per-link counter, which tracks traffic statistics and whose value is exported to the software layer through a small ISE. Compared with the solution presented in [29], here we also describe a high-level software interface, which can easily be mapped on concurrent execution models offered by high-level languages. Specifically, better use of data and computation locality is achieved by exploiting data-driven PXMs. The minimalist set of additional instructions is also used to dynamically control the configuration of each router, thus allowing the software to define customised topologies and partition the chip resources. It is worth noting that this group of instructions have been inspired by other works [16,30,31]. Here, we provide effective implementation of the physical substrate, thus further demonstrating the benefit of mapping data-driven execution paradigm over a highly configurable massive parallel processing fabric. All of these elements contribute to designing a fully scalable and programmable (software defined) NoC architecture.

## 3. Network-on-Chip Architecture

Our proposed NoC architecture is based on a hierarchical organisation: small rings serve more frequent local communications among a restricted group of PEs, while a global 2D-mesh interconnection provides connectivity for distant PEs. This hierarchy shows a better performance-to-power ratio and scalability when compared to the flattened designs (i.e., a single type of interconnection serving all the PEs in the chip). The introduction of reconfiguration capability in the proposed NoC architecture requires some additional component that we describe in the following. To limit the complexity of the router and ring-switch micro-architectures, we carefully designed these components, as well as adopting a communication protocol that takes advantage of less frequent transmission of a specific data packet payload, thus reducing the amount of used bandwidth.

### 3.1. Router Micro-Architecture

The primary component of the interconnection subsystem is represented by routers, which steer traffic from source to destination nodes depending on the information carried by data packets. In an NoC, packets are formed by a sequence of transfer units called *flits*. Depending on the complexity of the interconnection and communication protocols, packets may contain one or more flits. To correctly work, flits in an NoC must traverse the network in the same order as they leave the source node, thus allowing routers to correctly process all the data packets.

Our mesh router architecture resembles that of traditional designs, where input channels are connected to the internal crossbar switch front-side, which transfers the flits to the selected output channels. Small first-in first-out (FIFO) buffers, placed at the input and output channels, provide temporary storage for the in-flight flits. Such buffers also avoid incoming flits dropout every time the output channels are busy. The depth of these buffers depends on the average traffic conditions and may differ between input and output channels (typically, output channel buffers are smaller). In a traditional 2D-mesh router, five input–output channels are presented, referred to as east, west, north, south and local PE links. Since our architecture is based on a hierarchical topology, the router micro-architecture has been modified to include the eight input–output channels. Specifically, four out of eight channels are used to manage traffic within a tile (across ringlets); the remaining four channels allow the steering of the traffic among the tiles.

**Figure 3:**
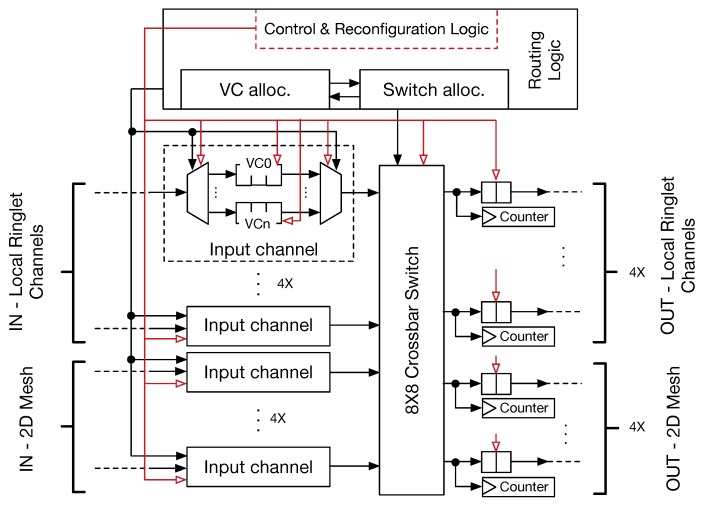
Internal micro-architecture of the 2D-mesh router connecting up to four ringlets. A router with all of the ringlets connected will form a physical tile. Red arrows are the power-gating signals.

Figure 3 represents our proposed router micro-architecture. The purpose of virtual channels (VCs) in a conventional NoC is twofold: (i) VCs are introduced to differentiate traffic flowing through the routers; (ii) they contribute to avoiding deadlocks when standard routing protocols are used (e.g., *x*–*y* dimension order routing (DoR). VCs are implemented by introducing multiple FIFOs in each input channel. Despite the higher flexibility in managing network traffic, VCs add complexity to the router control logic. In fact, it has to drive a two-stage selection policy: (i) input channels are arbitrated to select one of the VCs (e.g., using a round-robin (RR) strategy); (ii) input channels are arbitrated to transfer flits to the output channels. These two steps are managed by the VC allocator (VA) and the switch allocator (SA) modules. SA may be implemented with different technologies. However, the use of a tree of multiplexers allows us to dynamically map the selected input port with the corresponding output one, and to support reconfiguration of the NoC. Jerger et al. [32] showed that single-flit packets represent the large segment (up to 80%) of the network traffic for real applications (PARSEC benchmark). Aiming at lowering complexity with better performance of the router and ring switch control logic, we optimised the router (and consequently the ring-switch) micro-architecture for single flit data packets.

Furthermore, taking into account the recent growing interest in the ML or DL applications, we set the data packet length to 43-bits. Thus, a packet has 32-bits to transport data payload, and 11-bits to carry header information. Unlike scientific workloads, ML/DL applications use compact data types with a restricted precision (e.g., single-precision floating point, half-precision floating point, integer, custom data types). Our design choice has been influenced by such data type requirements, as well as from the fact that increasing the packet size leads to a quadratic increment of the internal crossbar switch area cost. To this end, we opted for a packet size that was as small as possible [33].

Every time a new packet is received, the corresponding header enables the routing logic to compute the correct output port to reach the destination. At the same time, VA and SA modules select which (packet) flit has to move towards the destination port. Aggressive speculation techniques can be implemented to maximise the network throughput. Such techniques allow VA and SA operations to be overlapped. The exploitation of speculative routing allows restricting the entire packet transfer to a single cycle. To this end, the following design choices have also been made: (i) use of store-and-forward mode; (ii) architectural optimization for single-flit packets with a short length; and (iii) use of speculative allocation techniques.

Router reconfiguration (see Section 4) is enabled by augmenting the routing control logic with specific hardware structures. Specifically, combinatorial logic is added to detect and parse configuration and control packets, as well as dedicated hardware tables and counters are used to keep information on virtual tiling and collect traffic statistics. This additional logic is also able to eventually turn-off (via PG hardware blocks, see red arrows on Figure 3) the internal router’s structures whenever applications do not use them, thus contributing to saving power. Performance counters are coupled to the output channels so that they are incremented by one every time a new flit is transmitted through the link.

### 3.2. Data Packet Structure and Control Flow

**Figure 4:**
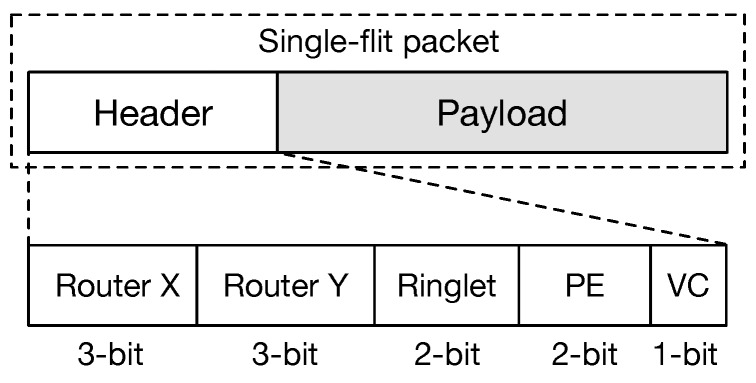
Structure of the single-flit packet.

The design has been optimised for single-flit packets, and for higher scalability (up to 1024 PEs). Flits have a length of 43-bits. They are formed by a header 11-bits long and a payload of 32-bits. The header structure allows for routing the flits within the global 2D-mesh hierarchically and within the ringlets in a tile. We have incorporated the xy-DoR protocol in our design. Here, each flit is moved on the *x*-dimension first, and then along the *y*-dimension, in order to reach the destination. To support the xy-DoR, the flit header contains two fields, each 3-bits long. In such way, a regular mesh of up 8×8 routers can be created. Within a physical tile, the ringlets are numbered progressively from 0 (the top-left ringlet) to 3 (bottom-right). Thus, a 2-bits field is used to select the destination ringlet. Similarly, PEs within a ringlet are numbered progressively from 0 to 3. Another 2-bit field is used to select the final destination. Finally, a 1-bit field is used to control the virtual channel assignment. The source router does the assignment of the VC. Aiming at separating traffic generated by applications running on the chip and controlling traffic (i.e., configuration packets and statistic requests—reading links’ counters), the latter is assigned to VC 0 by default. This design choice also contributes to simplifying routing and control logic. Figure 4 shows the structure of the single-flit packet used by our proposed NoC architecture.

The adoption of single-flit packets allows us also to simplify the control flow mechanism. Routers’ logic manages control signals used to enable packet transfer. This logic uses a backpressure mechanism, meaning every time the router has to send a packet, it raises a request signal towards the selected next hop. In case the selected router/ring station (next hop) has no free slots in the input queue to store the incoming packet, it resets the acknowledge signal. Resetting the acknowledge signal allows for temporaryily stopping transmission of packets at the source node. The complexity of such logic is reduced, although it ensures correctness and deadlock avoidance. Similarly, ring switches use a backpressure mechanism to control traffic within the bi-directional rings.

### 3.3. Ring Switch Micro-Architecture

Ringlets match well with communication patterns deriving from data and computation locality, and help with hiding the interconnection latency. Better exploitation of data and computation locality is also obtained by introducing the data-driven program execution architectural support. In such PXMs, threads belonging to the same task are grouped (hereafter, threads are intended to be composed of few tens of operations). They preferably are spawned on PEs belonging to the same ringlet. In this context, ring arrangement of the local interconnection permits a high-speed and low latency data exchange, with the communication that eventually can be extended to the other ringlets on the same physical tile. The combination of a simple control logic and the small ring diameter (we restricted to four PEs the number of nodes in a single ring) helps in reducing the communication latency. A data flit elapses one cycle to traverse each PE. Similarly, routers require only one cycle (using speculation) to forward flits. From this viewpoint, completing a transaction (i.e., reading a data from a remote PE scratchpad or writing data to) on a physical tile would require not more than 12 cycles. Thus, a smart distribution of the workload may reduce to few cycles the communication latency in average. Since ring switch logic is simpler than router control logic, further reduction of the latency can be achieved by using higher clock frequencies. For instance, the authors in [34] presented a ring-based NoC architecture where ring stations were clocked at the frequency that was higher than that of the connected PEs.

Ring switches are the basic blocks to implementing the proposed hierarchical NoC design. A ring switch (RS) forwards traffic over the ring. When the RS is located at the destination (source) of a transaction, the RS logic ejects (injects) the traffic from the global 2D-mesh (injects the traffic generated from the local PE). A ring unit (RU) is formed by the RS connected to the local PE. Only one RU in a ring connects to the 2D-mesh router. Such a unit is referred to as the ring-master unit (RMU).

**Figure 5:**
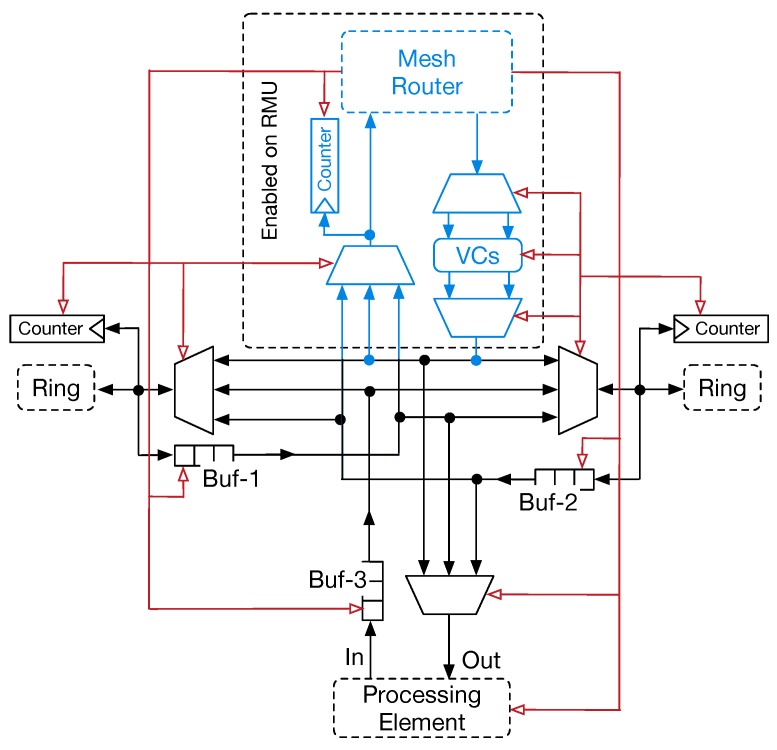
Internal micro-architecture of the ring-switch (RS). The link with the router is enabled only on the ring-master unit (RMU) (blue arrows and blocks). Red arrows represent the power-gating signal.

Figure 5 depicts the micro-architecture of RSs. The primary element is represented by a component that acts both as a multiplexer (MUX) and a demultiplexer (DMUX). We refer to this component as MDX. Two MDXs manage the traffic flowing in the ring in both directions, and are coupled with a small FIFO buffer to temporary store flits to eject from the ring. Compared to router micro-architecture, the buffer depth is smaller. Aiming at improving the performance and avoiding the packet dropping, MDXs implement a traffic prioritisation algorithm where flits traversing the MDX in the same direction have higher priority over data flits that need to be injected. Traffic prioritisation also enables RS to work at high clock frequencies, since the control logic is simplified. Conversely, when clocked to the same clock frequency of the routers, ring stations consume less power. An additional MUX and a dedicated buffer form the interface with the local PE. Similarly to the others, the depth of this buffer can also be small. The ejection of the flits from the ring has a higher priority over the injected traffic. It allows for reducing pressure on the internal buffers. The switching control logic is simplified compared to the traditional router control logic and is based on an RR policy. The main drawback of the traffic prioritisation is the emergence of the possible situation of starvation for the low priority traffic, which potentially may not acquire the access to the output link. In fact, low priority flits might potentially wait indefinitely, without winning link arbitration. To avoid such a situation, we allow traffic coming from low priority queues to be injected in the onward link after a fixed amount of elapsed cycles. This mechanism is easily implemented as a slightly modified RR selection strategy, where moving from one selected input to another is weighted by the priority of the input.

In Figure 5, blue lines and blocks represent the portion of the switch micro-architecture devoted to interface the RMU with the 2D-mesh router. An MUX forms the interface used to inject the traffic into the global interconnection (i.e., to forward traffic in the router’s input port) and an input channel which stores the flits ejected from the global 2D-mesh. The MUX integrates the governing logic that allows for selecting flits to forward in the input channel of the router. It is worth observing that the RMU is interfaced with a dedicated input channel, thus preventing contention with other traffic sources on the router. This input channel resembles the structure of input channels used in the router micro-architecture. Thus, it provides the same number of VCs (i.e., to support multiple traffic classes and avoid deadlocks). Allocation of VCs is based on the RR strategy, which avoids buffer exhaustion and traffic starvation. Similarly to the PE interface, traffic injection in the router has higher priority, since this reduces pressure on the ring buffers. A weighted RR allocation scheme is used to avoid starvation of the flits.

The presented micro-architecture significantly contributes to the reduction of the overall area cost and power consumption of the interconnection subsystem. This advantage also derives from the possibility to remove router interface in three out of four RUs. When implemented on an application-specific integrated circuit (ASIC) device, additional low-level optimisation (e.g., using small node manufacturing technology may allow the reduction of the area consumed by the wires) can be made. Similarly to routers, RS can also be turned-off by enabling a PG signal (see red arrows in Figure 5). Such signal is generated by the control logic of the 2D-mesh router. The PG mechanism can control at the granularity of a physical tile for further wiring reduction. Thus, the corresponding signal is spread to all the ringlets belonging to the same physical tile. It is worth noting that, in this case, both the RS and the PEs are in PG mode.

## 4. NoC Adaptability

Dynamic hardware adaptation is a feature demanded by many applications to achieve an adequate level of performance. For instance, in deep convolutional neural networks (CNNs), the size of the filters used in each layer of the network can vary. Thus, an optimal mapping of such kind of algorithms requires the capability of the underlying hardware to be reconfigured. To support this, our solution leverages on an effective application software interface, which allows for control 2D-mesh router port mapping. The interface provides the application developer with the means for creating custom virtual topologies, as well as to better partition the physical resources of the CGRA (*virtual tiles*—VTs). VTs enable system-level software (e.g., run-time libraries, operating system, hypervisor) to reserve resources for multiple applications (or multiple tasks of the same application) running on the same chip, thus avoiding interference among them.

**Figure 6:**
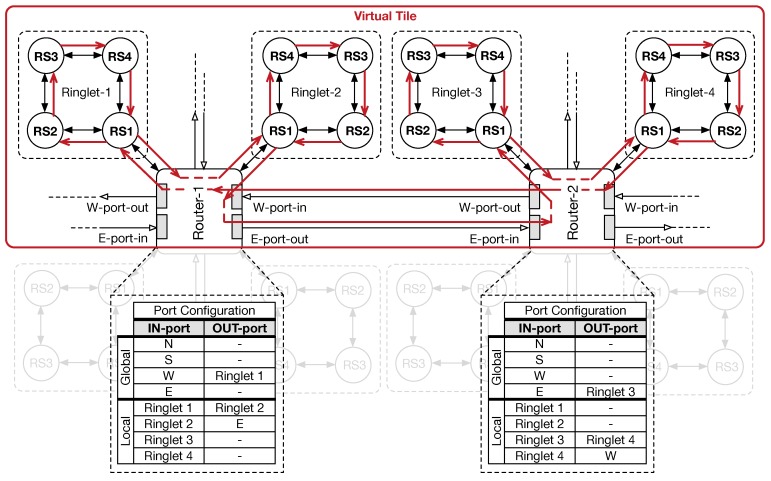
Coarse-grain reconfigurable architecture (CGRA) reconfiguration: two physical tiles (PTs) are reconfigured to create a single virtual tile (VT) using half of the ringlets in each PT—in red, the virtual topology arrangement.

Figure 6 shows an example of VT creation. Without loss of generality, the example shows how resources belonging to neighborhood physical tiles can be aggregated. To this end, two mesh routers share two ringlets each: red arrows in the figure shows how traffic, flowing in one direction, moves through the virtual ring that is created by aggregating four separated ringlets (VT). Reconfiguration requires that only routers must be configured. To ease router configuration, ring stations within a ringlet are numbered in such a way that RS1 is always the RMU. Through our software interface (see Section 5), internal crossbar switches are set in such way that traffic is maintained inside the virtual ring (i.e., the red path in Figure 6). For instance, Router-1 is configured as follows. West input port directly forwards traffic into the input buffer of Ringlet-1 (RS1 switch). Similarly, traffic ejected from RS1 switch (Ringlet-1) is steered in Ringlet-2. Finally, east output port receives traffic that flows away Ringlet-2. Like the case of Router-1, a similar configuration can be provided for Router-2, to inject/eject traffic from the local ringlets correctly. Through this configuration of Router-1 and Router-2, our solution allows the ringlet arrangement (within the VT) to resemble that of a global unidirectional ring structure. However, the configuration of routers can be further extended to support a bidirectional flow of the flits, as well as other type of topologies. It is worth noting that routers’ configuration is kept within an internal register, which is organised as a small table (see port configuration tables in Figure 6) mapping input ports with output ports on the crossbar switch. Such register provides 32-bits for mapping the configuration of local connections (i.e., the ringlets within a PT) and 32-bits for mapping global connections (i.e., the 2D-mesh). The register, 64-bits long in total, contains, for each table entry (1 byte long), a bit vector corresponding to the selection signals for the internal crossbar tree of MUXs. An additional 1-bit register, whose content is driven by dedicated instructions, allows for enabling or disabling the use of such ports mapping.

**Figure 7:**
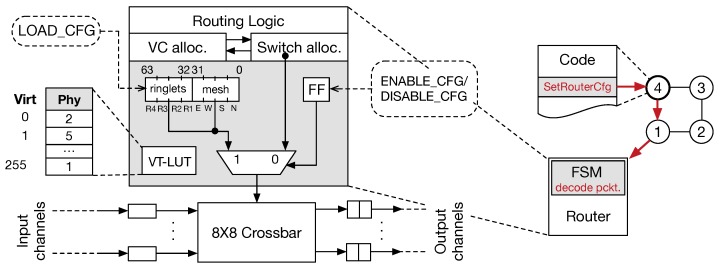
Router micro-architectural extension for supporting dynamic reconfiguration (**left**). An example of the reconfiguration flow is also depicted (**right**).

The proposed software interface for managing the router configuration consists of a minimalistic set of instructions executed by PEs (see Section 5). On the one hand, the payload of our data (packet) flit delivers control (reconfiguration) information to set internal structure of the routers correctly. On the other hand, we extended the router logic accordingly. The additional logic consists of a *configuration register* that is organised to resemble a mapping table, a look-up table resolving virtual-physical PE indexing within a VT, a 1-bit enabling register, and an MUX. Furthermore, as FSM governing the whole system is also added. Figure 7 (left) shows the additional structures included in the router’s logic to manage the reconfiguration phase. Router configuration can be achieved by sending two subsequent control flits, one packet delivering the configuration for local ringlets, and the other delivering information for the global 2D-mesh. A third control flit allows for setting/resetting of single bit enabling register.

Figure 7 (right) shows an example of the router configuration phase: once the PE4 on the ringlet executes the SetRouterCfg instruction, a corresponding control flit is generated and sent to the local router, where the FSM is used to decode it and enable the configuration (previously loaded). Again, the FSM can be easily substituted with a dedicated look-up table (LUT). With the aiming of making easier the configuration and use of VTs, PEs must be numbered progressively from 0 to K − 1 (where K is the number of PEs included in a VT). Thus, to support such dynamic PE renaming mechanism, a dedicate look-up table (VT-LUT) is integrated. This LUT allows mapping PEs’ physical identifiers (i.e., the PE unique identifier that is used to identify it across the whole fabric) with their virtual counterpart. Virtual PE identifiers allow software interface to ease the access and identification of the PEs during computations and communications. The entire map of the whole chip would require 1 K entries, with each entry being 10-bits long. To reduce the area and energy costs associated with the implementation of this LUT, we found that providing up to 256 PEs in a single VT is enough for supporting ML/DL algorithm mapping well. It implies that a minimum of four VTs are available when the dynamic configuration is enabled. The LUT provides, for each virtual identifier (ranging from 0 to 255) used as the LUT input, the corresponding physical identifier in the chip. Thus, the physical identifier is used by the router logic to manage the flit headers correctly. Dedicated instructions allow for managing the setup and reset of such LUT. To complete the configuration of the LUT, 86 control packets must be sent to the target router, with 5504 packets in total for the whole chip. An additional 128 packets are required as *starting* packets (see subsection in Section 4) to enable router configuration. Similarly, the crossbar configuration requires four packets per router, along with a starting packet and the command packet.

### Communication Protocol

The single-flit packet structure does not permit encoding in the packet header the information for issuing control signals to configure the network and read per link counter statistics. Thus, such information must be carried within the packet payload.

We adopted a communication protocol to avoid consuming bits by reserving part of the payload for control information. Here, the control packets are signalled by sending an initial *starting* flit. Since a payload with an all bits set (i.e., the payload is 0xFFFFFFFF) is rare, we use such value to enable the transmission of a subsequent set of control flits. Furthermore, a simple combinatorial logic can be used to detect such packets. Conversely, when such data flit must be transmitted to the destination, a pair of flits with the 0xFFFFFFFF payload is transferred. Preliminary simulations confirmed to us that the penalty is negligible, especially when real application traffic is taken into account. To deal with such protocol, the routing logic is augmented with simple FSMs that can process configuration commands or performance counter readings. Table 1 shows the flit sequences (packets) exchanged between the source and destination nodes. All of the sequences comprise the starting flit followed by the flit specifying the command. We used “don’t care” notation (X) to indicate content that depends on the selected destination target. This means that a group of bits in the command flit can serve as a mask to select the router or one of the RSs as the final target. The protocol ensures that configuration flits can be correctly delivered without sacrificing bandwidth for the application traffic. For instance, the internal router port mapping requires sending four consecutive flits, while the configuration of VT-LUTs requires up to 88 flits each (here, we also included the starting and command flits).

## 5. Software Interface

The ISA of PE is extended with a small group of instructions that serves the purpose of managing the reconfiguration phase, monitoring links’ traffic and supporting workload distribution. With the aim of keeping our interconnection agnostic with regards to the specific PEs (micro)-architecture (i.e., the processor word length, embedded local memories, cache hierarchy), a generic software interface (essentially the ISE) is presented. The interface is composed of instructions that can be conveniently wrapped by a function in standard high-level languages (e.g., Go, C/C++). Without losing generality, in the following, we assume a generic PE providing an in-order execution model, and embedding a small local scratchpad memory:

- SetRouterCfg ($RD, $RS): sends a topology configuration request (i.e., topology configuration specifies for each input port, which output ports can be used to forward the traffic) to the routers, by specifying the memory address where the configuration is stored ($RS), while register $RD specifies the destination router.
- SetRouterLUT ($RD, $RS): requires the internal routers’ look-up table (LUT) to be configured to expose the VT resources to the application. Routers use the internal LUT to expose PEs belonging to the same VT to the application, by numbering them sequentially (i.e., 0…K−1, where K is the number of PEs in the VT). The destination router is encoded in the $RD register, while register $RS specifies the memory base address where the router LUT content is stored.
- ReadCounter ($RD, $RS1, $RS2): reads the content of a link’s counter, by specifying the link to read. Performance counters can be read back from any PE, by specifying either the router or the ringlet-PE pair from which to read the corresponding counter. A sequence of control flits containing the counter data is sent back by destination router ($RD). The $RS1 register contains a mask specifying the link target, while source register $RS2 will store the value that has been read.
- ResetCounter ($RD, $RS): allows for resetting traffic statistics by specifying link performance counters to reset ($RD). The $RS register stores the mask used to select the counters (either routers or ringlet-PE pairs can be targeted).
- ResetRouterCfg ($RD): allows for resetting the current topology configuration. The target router is specified by the destination register $RD.
- ResetRouterLUT ($RD): allows for resetting the content of the internal router LUT. The target router is specified by the destination register $RD.
- EnableRouterCfg: allows for enabling routers’ configuration. Once the instruction is executed, a corresponding control flit is sent in a broadcast (actually, a sequence of control flits is generated, each targeting a router) to force the corresponding bit within each router to be set.
- DisableRouterCfg: allows for disabling the entire chip configuration (i.e., the physical chip fabric becomes accessible. Once the instruction is executed, a corresponding control flit is sent in broadcast (actually, a sequence of control flits is generated, each targeting a router) to all the routers, forcing their internal custom configurations to be discarded.

In addition to the eight instructions aforementioned, the following instructions are introduced to effectively support data-driven PXMs (i.e., tasks/applications composed of multiple concurrent groups of operations, here referred as threads, whose execution follows an explicit producer–consumer scheme):

- CreateThread ($RD, $RS1, $RS2): causes a new thread to be scheduled. To this end, the code pointer ($RS1), and where the unique thread identifier is stored ($RD) must be specified.
- ReadData ($RD, $RS1, $RS2): reads data ($RD) from the scratchpad memory. $RS1 and $RS2 are used to specify the base address and offset where to perform the read operation.
- WriteData ($RD, $RS1, $RS2): writes data to the scratchpad memory of another PE belonging to the same VT ($RD). $RS1 and $RS2 contain respectively the base address and the offset in the scratchpad where to perform the write operation. Unlike the read operation, the value to write should be stored in a fixed register (for simplicity we assume the first general purpose register available in the PE’s register file).
- IncreaseMem ($RD, $RS1, $RS2): requires the destination PE ($RD) to increase by one its local memory (scratchpad) location, whose base address and offset are stored respectively in $RS1 and $RS2 registers.
- DecreaseMem ($RD, $RS1, $RS2): requires the destination PE ($RD) to decrease by one its local memory (scratchpad) location, whose base address and offset are stored respectively in $RS1 and $RS2 registers.
- DeleteThread ($RD): marks the thread ($RD) as completed, allowing associated resources to be released.

### 5.1. High-Level Programming Interface

The software interface (ISE) presented in the previous section allows controlling, at a low level, the adaptation of the interconnection. A higher level programming interface is further required to ease the exploitation of the massive parallelism offered by our design. The capability of expressing parallelism directly in the program is mandatory. Starting from the observation that most of the ML/DL applications behave in a data-driven fashion, we introduce a high-level programming language interface. We implemented such interface within the Go language. Go provides an inherent concurrent execution model. We introduced a PXM, based on the producer–consumer paradigm which can reduce the effort for the programmer to describe concurrent activities in the application and ease the mapping on the proposed chip architecture. By forcing concurrent Go functions to exchange values only via dedicated data structures used for buffering input data (channels), the user can easily express parallelism in the application following an explicit data-driven scheme.

#### 5.1.1. Data-Driven PXM

PXMs specify the mechanism for synchronising threads. It is the process in which the memory is accessed and the threads are scheduled, created and destroyed on a target device. Unlike the von Neumann execution model, data-driven models require a private block of memory (*frame*) to store inputs used by the threads to run, a counter storing the number of inputs still not received (*scheduling slot*–SS), and the pointer of the thread body. The SS is decremented every time the consumer thread (here, we consider a thread as a sequence of operations atomically executed) receives a data from a producer thread. The triggering rule assumes that a thread is eligible for the execution once its SS counter is reduced to zero. In an explicit data-driven PXM, threads are allowed to explicitly schedule the execution of new threads, by adequately initialising their SS and passing the instruction pointer. We call threads adhering to this model data-driven threads (*DD-Threads*).

Go implements concurrent execution through the *communicating sequential processes* (CSP) mechanism. Such model is enabled by placing the *go* keyword before the name of a function that must be executed concurrently. The function becomes a *goroutines*. By default, goroutines execute independently from the others, once their input list becomes available. Channels, special input buffers (i.e., typed storage elements), can be included in the goroutine input list. Channels can also be declared as buffered. It means that it is also possible to specify the number of available slots. In that case, a read operation on an empty slot leads the goroutine, waiting until the value becomes available (similarly, a write operation on a full buffer). Goroutines provide an implementation of the proposed DD-threading model.

#### 5.1.2. Mapping Goroutines on DD-Threads

**Figure 8:**
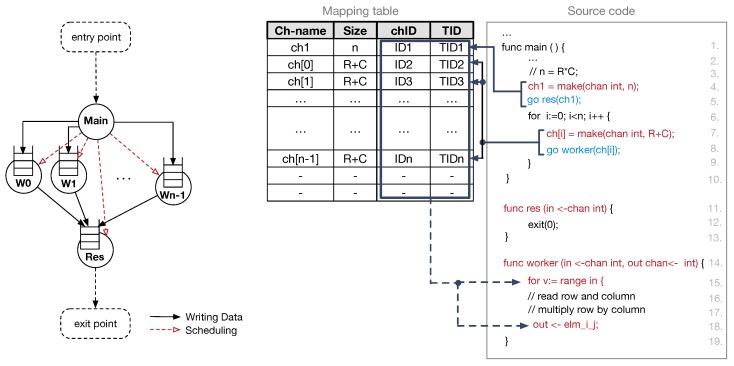
General matrix-matrix multiplication (GEMM) kernel written in Go (**right**). On the left side, the corresponding dataflow graph along with the mapping table.

Goroutines offer enough flexibility to easily map to data-driven threads (DD-Threads) for the execution. For this purpose, we use an illustrative example of the general purpose matrix–matrix multiplication (GEMM). It also takes inspiration from typical implementations of ML/DL algorithms. The Go code for this kernel is represented in Figure 8 (right) along with the corresponding dataflow graph–DFG (left). The dataflow graph is useful to visualise how the whole computation split among different concurrent tasks, and how such tasks synchronise each other. In Figure 8, red dashed arrows represent scheduling operations, while solid black arrows define data dependencies between producer and consumer DD-Threads. Such data dependencies are at the basis of the synchronisation mechanism exploited in the proposed explicit data-driven PXM.

The compiler is responsible for mapping goroutine calls to DD-Thread scheduling operations. First, during the analysis of the source code, the compiler collects information needed to allocate channels in the frame memory blocks. Since the thread communication process resembles a producer–consumer scheme, the channel allocation can be done either in the producer frame memory or in the consumer frame memory. Both producers and consumer threads have access to a PE’s local scratchpad memory to manage their frames. However, our data-driven PXM allows multiple DD-Threads to produce input values for a consumer thread, thus suggesting that the correct choice is the channel allocation in the consumer frame memory block. Furthermore, all the read operations are performed on the local memory, thus reading channel data from the local scratchpad speed up the execution of runnable threads. Therefore, we enforce the compiler to allocate buffers in the consumer memory.

The left side of Figure 8 shows the result of the allocation of channels: each node of the graph, which represents a consumer DD-Thread, has an associated input queue. It is worth noting that the process includes the decoupling between a channel declaration and its actual allocation in the frame memory block. The channel is declared every time the *make* keyword is encountered in the code. This keyword allows the compiler to keep track of the channel allocation requests (i.e., the allocation of the channel and the number of slots to be reserved). The number of slots in the channel defines the initial value of the SS counter. Specifically, the compiler acquires a unique identifier for the channel (i.e., chID = get_chID (channel_name)), which also identifies the consumer PE. This identifier is registered in an entry of the mapping table managed by the compiler. The table allows mapping functions and channel names with their actual unique identifiers. The first time the compiler encounters the consumer function (e.g., line 5 and line 8—Figure 8), the associated *go* keyword is mapped by the compiler into a request for a new unique thread identifier (i.e., TID = get_tID (chID, size)—it also identifies the thread frame within the assigned PE) and a thread scheduling request (i.e., schedule (function, chID, TID)—it copies the instruction pointer of the thread body in the destination PE). The right side of Figure 8 depicts an instance of the mapping table.

As previously mentioned, channel reading operations are always performed on the local memory. The semantics of such operations is ensured by the compiler, by substituting the channel name with the corresponding channel identifier (chID) which is obtained through a look-up in the mapping table. The substitution is performed every time the compiler finds the definition of a goroutine. Similarly, code involving read operations is substituted with the corresponding data = read (chID, offset) function. To adhere with the data-driven PXM, the actual read requires specifying the offset of the target location within the frame. Write operations are mapped into corresponding write (chID, TID, offset, data) operations. Similarly to the case of reads, the compiler substitutes the channel name with the tuple formed by the channel identifier (chID) and the DD-Thread identifier (TID).

The compilation phase ends with all the information gathered except the actual identifier of the PEs that will execute the scheduled threads, while the mapping table can be discarded. Missing information (i.e., the identifier of the PEs) is acquired at run-time, every time the get_chID(), get_tID(), schedule(), read(), and write() functions are executed. Specifically, channel and thread identifiers are combined to select the target PE. The application entry point is represented by the instantiation of the *main* thread. Without loss of generality, PE 0 within the VT is always selected for the execution of the main thread. Instead, scheduling the other threads requires to properly combine the chID and the TID to obtain the unique identifier (i.e., $peID_{dst}$) of the PE that will execute the thread. To this end, we allow to use an effective hash function, as follows:

$$H\left(\cdot \right)\to \{peID_{dst}=\left(chID⊕TID\right)\;mod_{K}\}.$$

The ⊕ is the concatenation operator, and the *K* is the total number of available PEs in the VT. At this point, it is possible to transfer the execution context (i.e., the TID, the chID, the channel size, and the pointer to the thread code) to the destination PE (i.e., $peID_{dst}$). In addition, read and write operations require determining the destination PE. Therefore, the same hash function is applied. In [31], the authors show a way to accelerate the generation of unique thread identifiers, along with an effective way for performing thread scheduling in hardware, by adding a simple extension on the router logic. Such mechanism can be easily incorporated into our design.

#### 5.1.3. Linking NoC Software Interface

All the above-mentioned operations can be easily mapped on the set of instructions described in Section 5. Specifically, the six instructions introduced in the ISE to ease the scheduling and the control of threads running on the PEs can be exposed by an OS run-time library, which is used by the Go compiler to perform the mapping with the mentioned operations. For instance, data = read (chID, offset) maps directly on the ReadData ($RD, $RS1, $RS2) function exposed by the run-time library, by assuming data = $RD, chID = $RS1 and offset = $RS2.

## 6. Evaluation

We evaluated the effectiveness of the proposed NoC architecture regarding performance, scalability, area cost and power consumption. We performed our evaluation, by synthesising the proposed interconnection using an FPGA device. The analysis of the proposed programming interface (i.e., the instruction set extension and the high-level programming interface) along with the evaluation of its effectiveness has been evaluated in [30].

All of the experiments were performed on a Xilinx Kintex-7 ultra-scale device and using the Xilinx Vivado 2017.4 design suite to simulate, synthesise, and perform the place-and-route operations. The entire synthesis and placement process was performed considering the instantiation of both a variable number of 2D-mesh routers (from 1 to 64) and ringlets (ranging from 1 to 256). A single FPGA device does not offer enough resources to accommodate the largest configurations. Therefore, the evaluation of scalability and performance of such complex configurations (up to 1024 PEs), was performed on multiple FPGA devices connected through dedicated external high-speed links. To this end, the latency of such links must be scaled to reflect the same latency of intra-chip interconnections. Correct management of the traffic required input ports in the router to have two VCs. Routers implement an RR strategy and employ the xy-DoR to reduce the latency for reaching the output port while keeping the complexity of the control logic low. The router operates with a four-stage pipeline, which can be optimised by integrating speculative logic to reduce the number of cycles required to forward flits.

We evaluated the throughput and average packet latency for a configuration with multiple physical tiles by simulating the injection of uniform random traffic, since this traffic pattern allows for better capturing the average network behavior. Experimental results show the capability of the proposed design to scale very well: average latency increases from 90 cycles (16 PEs) to 220 cycles (1024 PEs), while the throughput increases from 12 packets/cycle (16 PEs) to 570 packets/cycle (1024 PEs).

### 6.1. Network Performance

**Figure 9:**
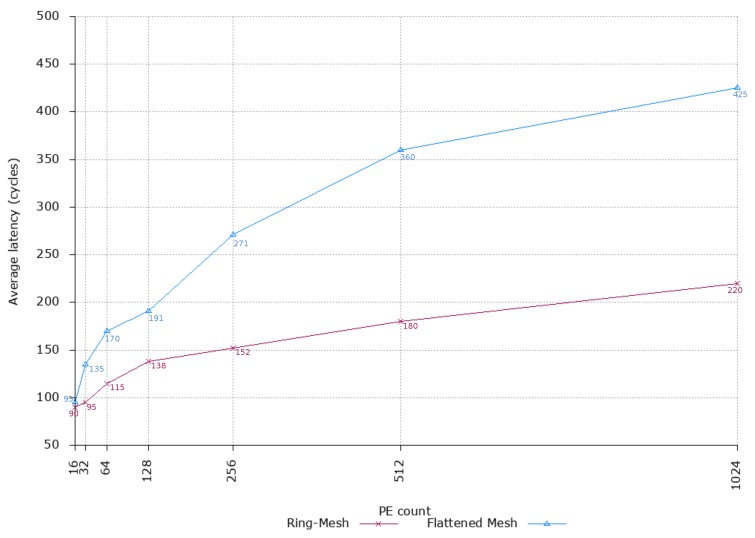
Average packet latency with increasing network size.

To analyse the scalability of the proposed design, we performed a set of experiments explicitly aimed at evaluating the average flit latency and throughput with increasing the number of PEs in the network. For comparison purposes, we set the injection rate at $I_{r}=0.75$, thus capturing the behaviour of the network under average traffic conditions. Figure 9 shows the average latency of the proposed NoC architecture compared with a flattened 2D-mesh architecture (i.e., all the PEs are connected through a 5-port router). Specifically, experimental data points are listed for both the designs. From reported values, it is clear that latency grows faster with the flattened design, almost doubling with network configurations containing more than 256 PEs.

Such behavior is well highlighted in the plot (Figure 9), where it is evident that the proposed NoC design performs with a significant reduction of the average latency up to 1024 PEs. Specifically, moving from a configuration with 16 PEs to that counting 128 PEs, the latency increases from 90 to 138 cycles. Conversely, for the same two configurations, the flattened 2D-mesh topology shows an increment of the latency from 95 to 191 cycles. For the next two network configurations (i.e., 256 and 512 PEs, respectively), the latency increment exhibited by our design is better (e.g., the increment from 128 to 256 PEs shows a lower slope of the curve), while, in the flattened 2D-mesh, the latency increment still follows a linear trend, thus showing worse performance. Finally, considering the largest configuration (i.e., 1024 PEs) the trend is still nonlinear for the proposed design and the latency drops to 220 cycles. Conversely, flattened 2D-mesh increases the latency up to 425 cycles (1024 PEs). If we combine these experimental observations with the lower power consumption and resource utilisation exhibited by our design, we can advocate that it scales better than conventional architectures. Thus, our design can be a good candidate for supporting next-generation high-performance many-core DL accelerators. Interestingly, to have similar performance using the flattened 2D-mesh topology, more resources are needed (e.g., larger number of VCs, deeper buffers), leading to a more power hungry and area consuming solution (similar to the cases reported in [35,36,37]).

**Figure 10:**
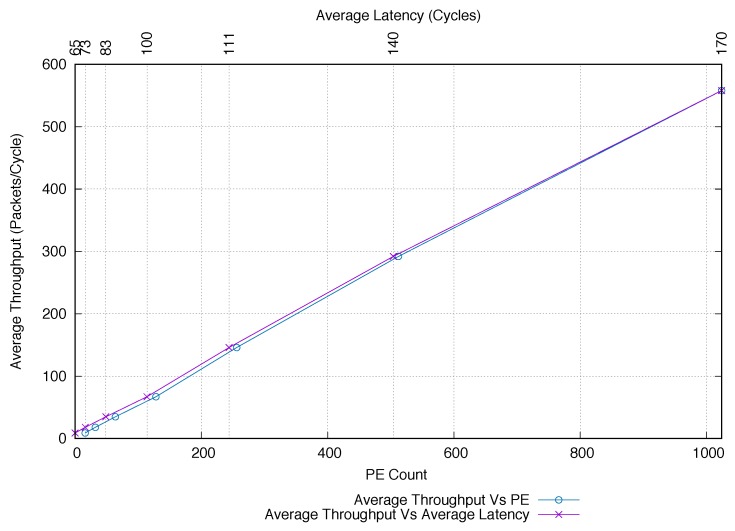
Comparing average network throughput and average packet latency with increasing network size.

To further confirm the capability of our design to scale well, we also analysed how the average network throughput improves with the growing network size. The results of this experiment are shown in Figure 10. It is evident that the average throughput tends to increase almost linearly by a factor of ≈2× when the number of PEs is doubled. For instance, for a physical tile (i.e., 16 PEs), the average throughput is 9.8 packets/cycle while it increases to 17.13 packets/cycle for 32 PEs. Similar behavior is observed when moving from 128 PEs (69.25 packets/cycle) to 256 PEs (147.7 packets/cycle), as well as when moving towards the largest configuration (i.e., from 512 PEs (288 packets/cycle) to 1024 PEs (570 packets/cycle)). The flattened 2D-mesh topology shows similar behaviour for lower PE counts. It is important to highlight that the average throughput is always lower than our proposed design and quickly starts to decrease when the PE count started to increase (i.e., for more than 128 PEs, our ring-mesh combination far outperforms the flattened 2D-mesh topology). Finally, we compared how the network performs when increasing the number of PEs, by plotting the average throughput versus the average latency. The result of this comparative analysis is also reported in Figure 10. From the plot, it is evident that our design provides an almost linear behaviour by keeping almost constant the ratio between average throughput and latency. It is worth noting that good performance exhibited by our design derives from the combined adoption of the single-flit packet format (packets require less time to be processed), and the traffic decoupling between ringlets (local traffic) and routers (global traffic).

### 6.2. Area Cost and Power Consumption

The area cost and power consumption of the proposed solution (for ringlets and mesh routers) are evaluated by measuring the consumed FPGA-device resources during the synthesis and place-and-route process.

**Figure 11:**
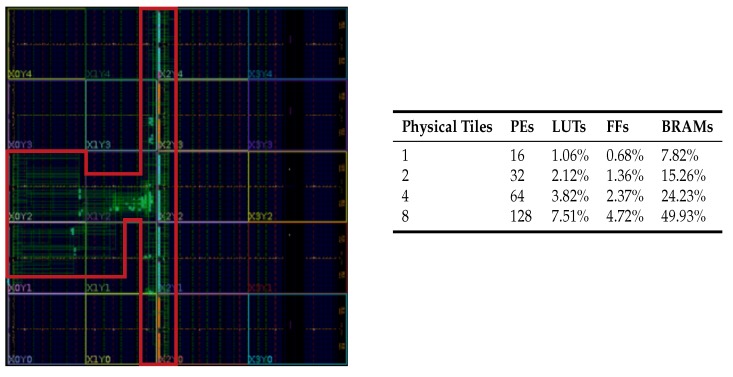
Result of the place-and-route process for a single physical tile (**left**)—the red box highlights the area of the FPGA device where resources are used). The synthesis results for different configurations are also represented (**right**)—single FPGA device.

We report the consumed resources regarding the percentage (i.e., the ratio between the used resources and the available ones on the FPGA). Here, we monitored the number of look-up tables (LUTs), registers (FFs), and block RAMs (BRAMs—each 36 Kbits in size). It is worth noting that presented results are intended for NoC configurations that can be instantiated on a single FPGA device. In fact, complex configurations (counting more than 128 PEs) have been implemented on multiple devices. Results allow us to advocate that the proposed solution is effective also regarding area cost. Interestingly, the implementation of 16 RSs (i.e., four ringlets composing a physical tile) requires less than 1% of the whole FPGA resources (except for the BRAMs). On the other hand, scaling up to 128 PEs consumes less than 50% of the BRAMS and less than 10% of LUTs and FFs. The place-and-route result for the 4×4 array of PEs (i.e., 4 ringlets and a router) on the FPGA device is depicted in Figure 11 (left). The complete synthesis/place-and-route results for different configurations (ranging from one to eight physical tiles) are reported in Figure 11 (right). Interestingly, the proposed design does not consume any of the available integrated DSPs. Furthermore, the number of used clock buffers is constant, irrespective of the configuration and it is below 1%. Such approach leaves enough space for the PE logic to explore more aggressively the available DSPs (e.g., in DL applications, DSPs are primarily employed to perform multiply-and-accumulate operations efficiently).

**Figure 12:**
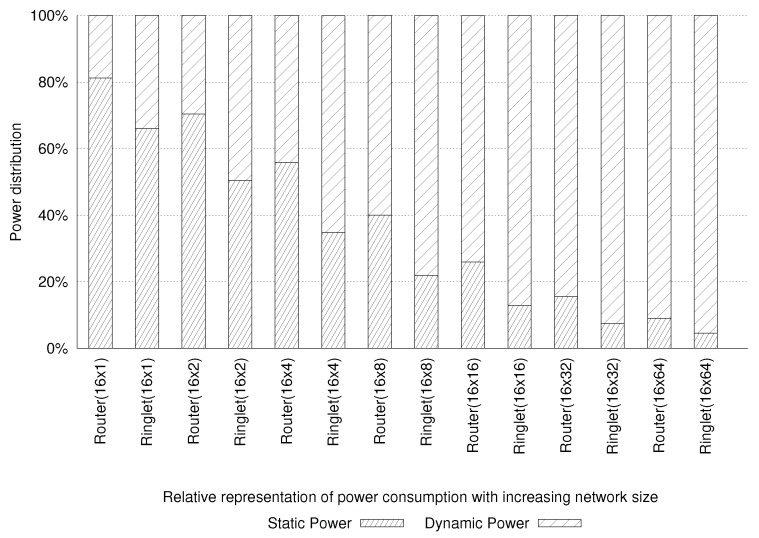
Static and dynamic components of the SDNoC power consumption.

Figure 12 reports both the static and the dynamic components of the power consumption for various NoC configurations. It can be seen that the data are reported for configurations counting up to 1024 PEs. When small configurations are considered (i.e., up to 64 PEs), the static component dominates the power consumption of the system (due to the leakage effects). Such effects result in static power consumption of the entire device. The dynamic component starts to dominate the total power consumption while moving to the larger configurations (i.e., more than 64 PEs). We also found that the static power consumption, for a physical tile, equals 806.0 mW (324.0 mW ascribed to the router and 482.0 mW to four ringlets). Conversely, the dynamic power consumption for the mesh router is only 75.0 mW, while the ringlets consume up to 3.502 W. The proposed design keeps its efficiency also using a large configuration since the dynamic power consumption does not increase linearly with the number of active PEs. It leaves enough space in the power budget for integrating PEs’ logic. For instance, configuring the network to use eight physical tiles (i.e., using 128 PEs) results in dynamic power consumption of 5.683 W, while static power consumption rises to 3.789 W. All of these results demonstrate the effectiveness of the proposed design as the primary interconnect subsystem for large CGRAs.

## 7. Conclusions and Future Work

The design of a many-core based accelerator for ML/DL applications requires improvements in all levels of the system (from programming interface to chip micro-architecture level). In this context, the interconnection subsystem plays a critical role, since it is required to efficiently exchange information (data packets) among a large number of PEs. In this paper, we propose a highly efficient interconnect fabric, whose architecture is based on a modular, hierarchical approach. To this end, we leveraged on the combination of ring-based interconnects to better serve local traffic exchanged among PEs placed close to each other, while global traffic (i.e., traffic exchanged among PEs that are far away from each other) is managed by a 2D-mesh interconnection. The advantages of the combination of different topologies are many-fold. Firstly, the performance is better than those shown by a flattened 2D-mesh design. Secondly, area and power consumption are greatly reduced. Finally, it better supports PXMs that are generally at the basis of ML/DL applications’ domain.

Here, we proposed integrating a reconfiguration feature into our lightweight modular design approach, allowing local and global traffic to be decoupled. A software interface is also proposed to easily exploit the advantages offered by our design. The low-level instructions executed by PEs allow controlling the network topology, its reconfiguration, and also the data packet communication. Next, a more abstracted interface is also explained: a high-level programming language is used to support a producer–consumer execution scheme, which directly maps to the features exposed by the underlying NoC architecture. Later, the preliminary evaluation confirms the advantages of our interconnection architecture.

Future work directions comprise a more in-depth performance analysis of the proposed interconnection fabric using different traffic patterns (e.g., bit reverse, transpose) and multiple injection rates. A detailed comparison with other topologies (e.g., fat-trees, 2D-torus) will also be the part of our future research work. It will also be very interesting to study the ML/DL application behaviour running on such hybrid NoC subsystem.

## Figures and Tables

**Table 1:** Configuration packet sequences used to configure the router logic and to collect statistics. These packet sequences are generated as a response to the execution of configuration instructions.

Instruction	Packet Number	Packet Description	Packet Payload
	1	Starting	0xFFFFFFFF
SetRouterLUT	2	Command	0x00000001
	3–88	Configuration	0b00X ...X
	1	Starting	0xFFFFFFFF
SetRouterCfg	2	Command	0x00000002
	3–4	Configuration	0bXX ...X
	1	Starting	0xFFFFFFFF
ReadCounter	2	Command	0x0000XX03
	3–4	Reply	0bXX ...X
ResetCounter	1	Starting	0xFFFFFFFF
	2	Command	0x0000XX04
ResetRouterCfg	1	Starting	0xFFFFFFFF
	2	Command	0x00000005
ResetRouterLUT	1	Starting	0xFFFFFFFF
	2	Command	0x00000006
EnableRouterCfg	1	Starting	0xFFFFFFFF
	2	Command	0x00000007
DisableRouterCfg	1	Starting	0xFFFFFFFF
	2	Command	0x00000008
